# All-Inorganic CsPbBr_3_ Perovskite Nanocrystals Synthesized with Olive Oil and Oleylamine at Room Temperature

**DOI:** 10.3390/mi14071332

**Published:** 2023-06-29

**Authors:** Getachew Welyab, Mulualem Abebe, Dhakshnamoorthy Mani, Aparna Thankappan, Sabu Thomas, Fekadu Gochole Aga, Jung Yong Kim

**Affiliations:** 1 Faculty of Materials Science and Engineering, Jimma Institute of Technology, Jimma University, Jimma P.O. Box 378, Ethiopia; gwelyabt@gmail.com (G.W.); muabeme@gmail.com (M.A.); m.dhakshnamoorthy@gmail.com (D.M.); 2 Department of Physics, College of Natural and Computational Science, Mizan-Tepi University, Mizan P.O. Box 260, Ethiopia; 3 Department of Physics, Baselius College, Kottayam 686001, India; aparnathankappan@baselius.ac.in; 4 School of Energy Materials, Mahatma Gandhi University, Kottayam 686560, India; sabuthomas@mgu.ac.in; 5 Department of Materials Science and Engineering, Adama Science and Technology University, Adama P.O. Box 1888, Ethiopia; fekadu.gochole@astu.edu.et; 6 Center of Advanced Materials Science and Engineering, Adama Science and Technology University, Adama P.O. Box 1888, Ethiopia

**Keywords:** perovskite, supersaturated recrystallization, CsPbBr_3_, olive oil, LaMer model, nucleation and growth, solvent engineering, nanocrystals, quantum dot, ligand

## Abstract

Room temperature (RT) synthesis of the ternary cesium lead bromide CsPbBr_3_ quantum dots with oleic acid and oleylamine ligands was developed by Zeng and coworkers in 2016. In their works, the supersaturated recrystallization (SR) was adopted as a processing method without requiring inert gas and high-temperature injection. However, the oleic acid ligand for haloplumbate is known to be relatively unstable. Hence, in this work, we employed the eco-friendly olive oil to replace the oleic acid portion for the SR process at RT. Resultantly, we found that the cube-shaped nanocrystal has a size of ~40–42 nm and an optical bandgap of ~2.3 eV independent of the surface ligands, but the photoluminescence lifetime (τ_av_) and crystal packing are dependent on the ligand species, e.g., τ_av_ = 3.228 ns (olive oil and oleylamine; here less ordered) vs. 1.167 ns (oleic acid and oleylamine). Importantly, we explain the SR mechanism from the viewpoint of the classical LaMer model combined with the solvent engineering technique in details.

## 1. Introduction

Halide perovskites (HPs) have the general chemical structure of ABX_3_, where A (methylammonium (MA) CH_3_NH_3_^+^, formamidinium (FA) CH(NH_2_)_2_^+^, methylhydrazinium CH_3_(NH_2_)_2_^+^, aziridinium (CH_2_)_2_NH_2_^+^ and cesium Cs^+^) and B (lead Pb^2+^, tin Sn^2+^, and germanium Ge^2+^) are cations, and X (chlorine Cl^−^, bromine Br^−^, and iodine I^−^) is an anion [1,2,3,4,5,6,7]. When these HPs serve as a semiconducting layer for the electronic and optoelectronic devices, they share some common properties such as defect tolerance, large absorption coefficient, high dielectric constant, low exciton binding energy, long charge carrier diffusion length, high charge mobility, and tunable bandgap [8,9,10,11,12,13]. Hence, the state-of-the-art devices show the power conversion efficiency of ~26% for photovoltaic (PV) cells, and more than 20% quantum efficiency for light-emitting diodes (LEDs) [4,14,15,16,17]. Furthermore, based on the excellent aforementioned properties, HPs have been applied to lasers, photodetectors, X-ray/γ-ray detectors, medical imaging devices, biosensors, photocatalysis, and luminescence solar concentrators [18,19,20,21,22,23,24,25]. However, the organic–inorganic hybrid HPs have an intrinsic problem in thermal stability originating from the organic cations such as MA and FA in the perovskite structure [26,27]. For example, MABr decomposes at around 250–350 °C [27]. Hence, to overcome this thermal instability, all-inorganic HPs such as CsPbX_3_ (X = Cl, Br, and I) have been employed for perovskite electronics [27,28,29,30,31,32,33,34].

However, it is notable that all-inorganic Cs-based perovskites with a wide bandgap (*E_g_* > 1.65 eV) [35,36] also have some stability issues. First, the Goldschmidt tolerance factor (t) is $\left(r_{A}+r_{X}\right)/\left\{\sqrt{2}\cdot \left(r_{B}+r_{X}\right)\right\}$, in which $r_{A},\;r_{B},\;\mathrm{and}\;r_{X}$ are the radius of cation A, cation B, and anion X, respectively [37]. In the case of CsPbCl_3_, CsPbBr_3_, and CsPbI_3_, each tolerance factor is 0.87, 0.816, and 0.805, respectively [38,39]. Considering that the cubic phase could be stable in the range of 0.813 ≤ t ≤ 1.107 [37], CsPbI_3_ is structurally unstable among the cesium lead halides. Second, the Cs-based perovskites undergo a multiple polymorph transition, such as orthorhombic, tetragonal, and cubic, depending on the temperature and composition [40]. To be a thermodynamically stable cubic phase with high symmetry, the perovskite should stay at the temperature more than 47 °C for CsPbCl_3_, 130 °C for CsPbBr_3_, and 300 °C for CsPbI_3_, indicating that CsPbI_3_ is the most challenging compound in spite of its promising energy bandgap ~1.73 eV [41,42,43,44]. Note that CsPbCl_3_ and CsPbBr_3_ each have a bandgap of ~3.03 eV and ~2.25 eV, respectively [45]. Furthermore, it is notable that CsPbI_3_ undergoes a phase transition from the black γ-phase to the yellow δ-phase (i.e., a non-perovskite structure with *E_g_*~2.82 eV), resulting in an undesirable compound for perovskite electronics [46]. Third, according to Li and coworkers, the thermal stability of Cs-based perovskites is in the sequence of CsPbCl_3_ > CsPbBr_3_ > CsPbI_3_. This is because the structural change was observed at 500 °C for CsPbCl_3_, 400 °C for CsPbBr_3_, and 200 °C for CsPbI_3_, respectively, which is based on in situ X-ray diffraction experiments as a function of temperature in ambient conditions [47,48]. Hence, we notice that CsPbCl_3_ and CsPbBr_3_ are superior to CsPbI_3_ from the stability point of view. In this work, we may focus on the green-emitting CsPbBr_3_ perovskite semiconductor [49,50].

In 1958, Møller reported the structure and photoconductivity of CsPbX_3_ crystals (X = Cl, Br, and I) [28]. Then, after more than five decades, in 2015, Kovalenko and coworker invented a hot injection (HI) method for synthesizing CsPbX_3_ (X = halide or its mixture) colloidal quantum dot materials with oleic acid (OA) and oleylamine (OAm) surface ligands [51]. Then, after one year, Zeng and coworker invented another method called supersaturated recrystallization (SR) for synthesizing CsPbX_3_ nanocrystals (NCs) with OA and OAm at room temperature [52]. In 2019, Shi and coworker tried to replace OA with an edible ‘olive oil (OO)’ using the HI method [53]. Then recently, Jing and coworker tried to replace OA with another ligand called 4,4′-Azobis(4-cyanovalericacid) (CA) at room temperature because OA and OAm can be detached easily from the surface of CsPbX_3_ [54]. On the other hand, in 2022, Ray and coworkers proposed a new technique to synthesize CsPbX_3_ (X = I/Br) by modifying the ligand chemistry using olive oil [55].

For fabricating a relatively more stable cubic phase of CsPbX_3_, there are three methods: (1) the synthesis of NCs with high surface energies, (2) surface ligand engineering, and (2) composition engineering [51,52,53,54,55]. These methods have been commonly utilized for synthesizing CsPbX_3_ QDs. Hence, the colloidal CsPbX_3_ NCs have several strong advantages compared to its bulk counterparts. First, based on quantum size and confinement effect, the energy bandgap of QD materials is easily tunable resulting in the full-color spectrum coverage. Second, the NC–ligand complexes can afford versatile solution processes via colloidal chemistry. Third, the perovskite NCs can enhance their optical properties, resulting in a high photoluminescence quantum yield (PLQY) and a narrow full width at half maximum (FWHM) for color purity, which is desirable for lighting and display applications [51,52].

In this study, we tried to replace OA with OO to synthesize CsPbBr_3_ NCs at room temperature for which we adopted the SR method. To the best of authors’ knowledge, in the past, the OO ligand was tested only by the HI method [53], although OO is promising as a surface ligand for the mass production process in future. Hence, in this work, we employed this OO ligand for synthesis of CsPbBr_3_ NCs at room temperature. Note that SR is a type of ligand-assisted reprecipitation (LARP) technique, affording a simple, low cost, and a large production capability in ambient conditions. Furthermore, basically, SR is an antisolvent-mediated solution process, resulting in the nucleation and growth of perovskite NCs. Hence, the classical LaMer model [56,57] and solvent engineering (SE) [58,59] should be very important for addressing the SR mechanism in the synthesis of CsPbBr_3_ NCs at room temperature.

## 2. Materials and Methods

### 2.1. Chemicals

Lead bromide (PbBr_2_, 99.0%, AR chemicals, Delhi, India), cesium bromide (CsBr, 99.9%, Sigma-Aldrich, Darmstadt, Germany), oleic acid (OA, 98%, Sigma-Aldrich, Darmstadt, Germany), oleylamine (OAm, technical grade 70%, Sigma-Aldrich, Darmstadt, Germany), olive oil (OO, Nice, Kochi, India), hexane (≥97.5%, Sigma-Aldrich, Darmstadt, Germany), *N*,*N*-dimethylformamide (DMF, 99.5%, AR chemicals, Delhi, India), and toluene (≥99.5%, AR chemicals, Delhi, India) were used in this experiment. All chemicals were used without further purification.

### 2.2. Synthesis of CsPbBr_3_ Nanocrystals

In the SR method [52], the perovskite precursors and ligands, CsBr (0.2 mmol, 43 mg), PbBr_2_ (0.2 mmol, 73 mg), OA or OO (0.5 mL), and 0.25 mL of oleylamine, were dissolved into 5 mL of a polar DMF solvent and stirred using magnetic stirrers at RT for two hours. Then, 1 mL of the perovskite precursor solution was titrated into 10 mL of a nonpolar toluene antisolvent and stirred for 60 s. Then, the CsPbBr_3_ NC dispersion exhibited a green-light emission under 365 nm UV illuminations, confirming that the reaction was successful through the SR method. Then, this colloidal dispersion was transferred to a centrifuge tube for centrifugations, and it was centrifuged at 8000 rpm for 10 min. The supernatant was discarded, and the resulting precipitate was re-dispersed in the nonpolar hexane. Then, the final CsPbBr_3_ sample was dried under a vacuum oven at 60 °C overnight. Finally, the CsPbBr_3_ samples were kept for further characterization at RT.

### 2.3. Characterization

The structural properties of CsPbBr_3_ (OA and OAm) and CsPbBr_3_ (OO and OAm) were determined by the X-ray diffraction (XRD) measurements measured using the Rigaku mini flex-300/600 diffractometer (Tokyo, Japan). The irradiation used CuKα, *λ* = 1.5406 Å at 40 kV and 15 mA for the XRD analysis. The CsPbBr_3_ NC’s size and shape were determined by using the high-resolution transmission electron microscopy (HR-TEM) (Model: JEOL, JEM-2100) with an operating voltage of 200 kV. The optical absorption was characterized by using the Ultraviolet-visible (UV–vis) absorption spectroscopy (SHIMADZU UV-2600, Kyoto, Japan), whereas the photoluminescence (PL) spectra were measured using a spectrophotometer (SHIMADZU RF-6000, Kyoto, Japan) at the excitation wavelength of 375 nm. The Fourier transform infrared spectroscopy (FT-IR) analysis was performed by using the PerkinElmer Spectrum Two FT-IR Spectrometer. For this purpose, the attenuated total reflection (ATR) was employed as a sampling technique to record the FT-IR spectra of the drop-cast CsPbBr_3_ thin film in the range 4000–400 cm^−1^ with resolution of 4 cm^−1^. Here, for the drop-cast thin films, the glass substrate was washed in detergent soap, followed by ultra-sonication using distilled water, acetone, and isopropanol, respectively, for 15 min per each process. Then, the cleaned substrate was dried at 80 °C for 1 h in vacuum oven and subsequently cooled down at room temperature. The prepared sample solution (10 mg perovskite precursors in 1 mL of toluene) was drop cast on the clean glass substrate and dried in ambient conditions. The PL decay curves were recorded by using a time-correlated single photon counting (TCSPC) (Model: Fluorolog 3 TCSPC, Horiba, Irvine, CA, USA).

### 2.4. Computational Methods

The electronic band structures of the CsPbBr_3_ unit cell were calculated using the Cambridge Serial Total Energy Package (CASTEP, Materials Studio 2017) software with the density functional theory. The Perdew–Burke–Ernzerhof (PBE) parametrization of the General Gradient Approximation (GGA) was used to describe the exchange correlation functional. The unit cell in the Brillouin zone was applied to evaluate the electronic band structures. Energy of 5 × 10^−5^ eV/atom, maximum force of 0.01 eV/Å, maximum displacement of 5 × 10^−4^ Å, and maximum stress of 0.02 GPa were used for all geometry optimization.

## 3. Results and Discussion

**Figure 1:**
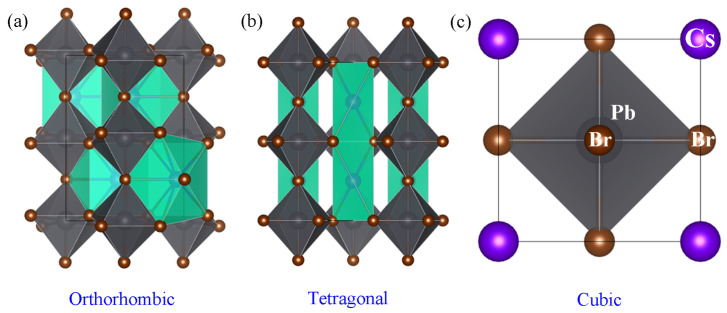
Polyhedral of CsPbBr_3_ unit cell: (**a**) orthorhombic, (**b**) tetragonal, and (**c**) cubic structure.

Figure 1a shows the orthorhombic structure (space group, Pbnm) of CsPbBr_3_ crystal. However, when temperature increased, CsPbBr_3_ undergoes a phase transition from orthorhombic to tetragonal (P4/mbm) at 88 °C and from tetragonal to cubic ($Pm\bar{3}m$) at 130 °C through a tiny rearrangement of the unit cell atoms [60,61]. On the other hand, in the case of NCs, the unit cell structure could be affected not only by crystal size/shape, but also by the Pb^2+^/ligand ratio [34]. For example, the cubic phase was observed at RT if the crystal size is in nanoscale, indicating the metastable state of NCs. In the case of the electronic structures of CsPbBr_3_ unit cell, the information could be found in the Supplementary Materials (SM), Figure S1, displaying that the bandgap (*E_g_*) is reduced with from 2.40 eV (cubic) to 2.31 eV (orthorhombic) via 2.37 eV (tetragonal).

**Figure 2:**
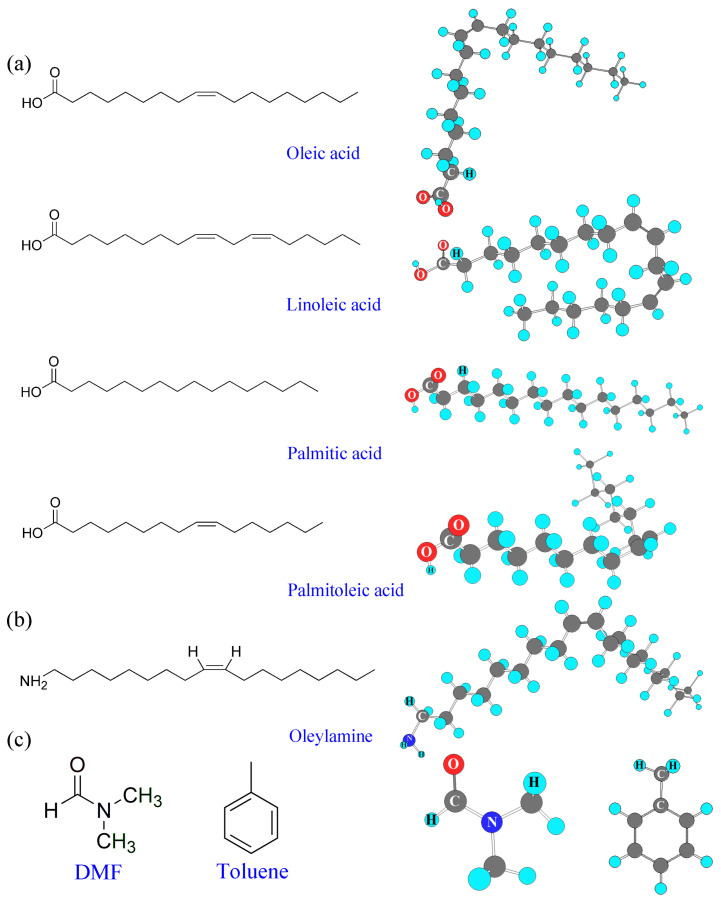
Chemical structures of (**a**) olive oil: glycerol fractions, ~98%; (**b**) oleylamine; and (**c**) DMF and toluene.

Figure 2 shows the chemical structure of (a) OO, (b) OAm, and (c) DMF and toluene, among which both organic OO and OAm surface ligands were employed as a mixed ligand for the synthesis of CsPbBr_3_ at RT using the SR process [52]. As shown in Figure 2a, the OO’s glycerol fractions (~98% of total OO) are composed of oleic acid, linoleic acid, palmitic acid, and palmitoleic acid, but it also contains small amounts of non-glycerol fraction (~1–2% of the total OO by weight), such as biophenols and triterpenic acids [62]. Here, because of the conformational freedom (e.g., a chain folding) of the C-C single-bond rotation, the high carbon ligand may undergo a chain folding in a typical solvent medium, DMF. Importantly, it is noteworthy that the compositional engineering (or mixing strategy) has been commonly used for perovskite electronics for enhancing stability, e.g., cation mixtures (MA^+^/FA^+^), metal ion mixtures (Pb^2+^/Sn^2+^ or Ge^2+^/Sn^2+^), halogen mixtures (Cl^−^/Br^−^/I^−^), perovskite dimensional mixtures (2D/3D), etc., which resulting in the enhancement of stability of materials and devices [62,63,64,65,66,67]. In the same vein, when we use the edible OO as a surface ligand, it might be useful in stability with a guaranteed benefit of eco-friendliness, which is a desirable characteristic for a mass production process in future.

**Figure 3:**
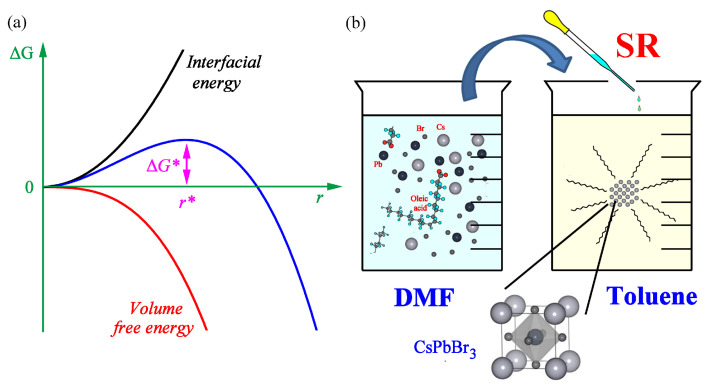
(**a**) The free energy change (ΔG) in the nucleation and growth process as a function of *r* (a radius of a spherical nucleus by assumption). Here, ΔG* is the critical free energy barrier at the critical radius (r*), where a unit cell is formed. (**b**) Schematic explanation of supersaturated recrystallization (SR) of CsPbBr_3_ in the antisolvent toluene.

Figure 3a shows the free energy change (ΔG) as a function of the radius (r) when the nucleus is assumed to be a spherical according to the LaMer model, a classical nucleation theory [56,57]. Here, the free energy change ($\Delta G_{hom}$) for homogenous nucleation and that ($\Delta G_{het}$) for heterogeneous nucleation could be defined as follows,

(1) $$\Delta G_{hom}=-\frac{4}{3}\pi r^{3}\Delta G_{v}+4\pi r^{2}\gamma_{SL}$$

(2) $$\Delta G_{het}=\left\{-\frac{4}{3}\pi r^{3}\Delta G_{v}+4\pi r^{2}\gamma_{SL}\right\}\cdot \frac{\left(2+\cos\theta \right){\left(1-\cos\theta \right)}^{2}}{4}$$

where $\Delta G_{v}$ is the free energy change associated with the formation of a small volume of solid, whereas $\gamma_{SL}$ is the solid/liquid interfacial free energy. Furthermore, $\Delta G_{het}=\Delta G_{hom}\cdot S\left(\theta \right)$, where $S\left(\theta \right)=\left(2+\cos\theta \right){\left(1-\cos\theta \right)}^{2}/4$ is a shape factor at a wetting angle θ. According to the LaMer model [57], when a solution is supersaturated by undercooling, a nucleus could be formed. If this nucleus is larger than the critical size (r*), the spontaneous growth is available because ΔG is decreasing as shown in Figure 3a (see the blue solid line). In the case of the SR process, a supersaturated condition is directly available when the perovskite precursor solution (a colloidal dispersion containing bromide plumbate, ${\mathrm{PbBr}}_{n}^{2-n}$ with n = 2–6) is dropped into the antisolvent medium, e.g., toluene with Gutmann’s number (*D_N_*) ~0.1 kcal/mol, i.e., Lewis basicity (see Table 1 for details) [68]. Here, it is noteworthy that a nonpolar toluene with solubility parameter, *δ* = 8.9 (cal/cm^3^)^1/2^ is antisolvent for the bromide plumbate but it is still miscible with the polar DMF solvent (*δ* = 12.1 (cal/cm^3^)^1/2^) [69], indicating that the perovskite precursor ions (Cs^+^, Pb^2+^, Br^−^) will be directly exposed to the antisolvent toluene molecules, resulting in the nucleation and growth of perovskite NCs as explained through the critical point (r*–G*) in the LaMer model. However, it is notable that in the case of SR process, the supersaturation was reached simply by adding the precursor solution into the antisolvent (not by undercooling like in the LaMer model). Interestingly, the SR process is similar to the well-known Solvent Engineering (SE) (i.e., one-step antisolvent method) in the sense that the wet precursor solution is directly exposed to the antisolvent molecules [58,59]. However, the difference is that the SE method is processed during the spin-coating step by dispensing the antisolvent on the top of a wet precursor solution. Furthermore, SE allows for the formation of intermediate phase when a polar solvent DMSO is employed, whereas SR may not allow for it because the colloidal dispersion may undergo a direct crystallization (at least without a co-solvent such as DMSO). Importantly, the SR process corresponds to the heterogeneous nucleation and growth of CsPbBr_3_ perovskite because the nanoscale crystallization was processed through the surface of haloplumbate dispersed in the DMF medium. Recall that the perovskite precursor solution is a typical colloidal dispersion [70], i.e., haloplumbate is dispersed in a solvent medium, herein DMF with *D_N_* = 26.6 kcal/mol. In the case of Br-, its *D_N_* is 33.7 kcal/mol [71], indicating that Pb^2+^ (Lewis acid) may have a stronger coordination bonding with Br^−^ ions (Lewis base) than DMF to form the acid–base adducts in spite that DMF is a good solvent for the perovskite precursor materials. See Figure S2 in the Supplementary Materials for the vials containing CsPbBr_3_ NCs in the supersaturated solution/dispersion.

**Figure 4:**
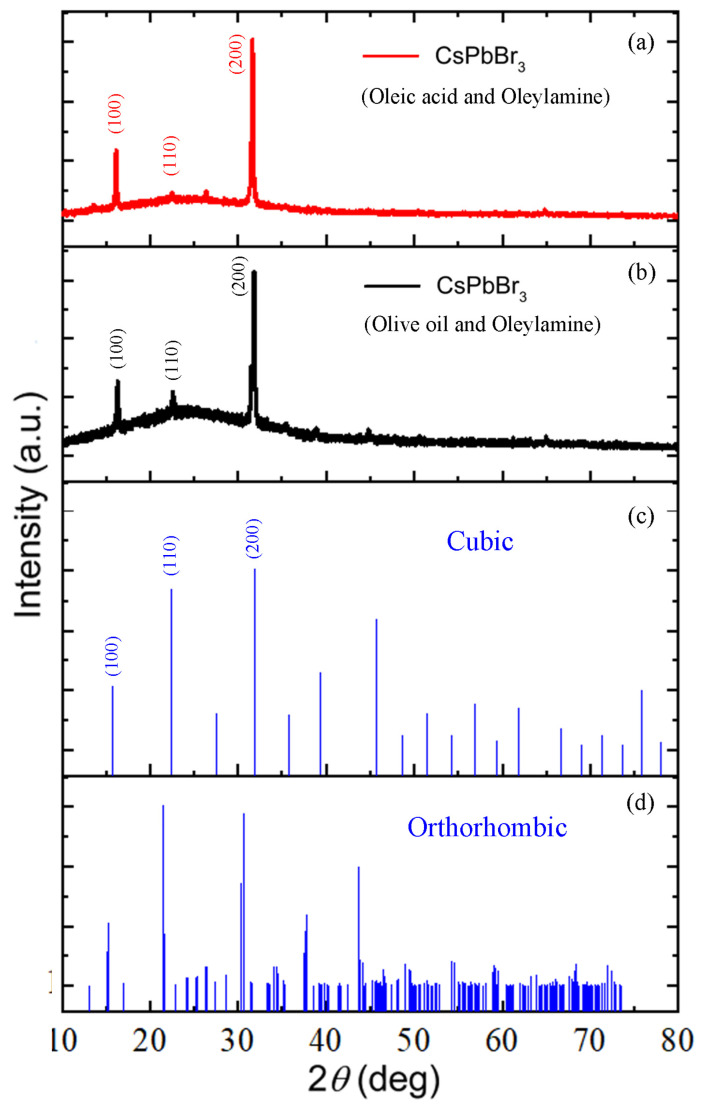
XRD patterns of CsPbBr_3_ nanocrystal drop-cast thin film depending on the ligand species: (**a**) oleic acid and oleylamine and (**b**) olive oil and oleylamine. (**c**) Joint Committee on Powder Diffraction Standards (JCPDS) (PDF#96-153-0682) and (**d**) JCPDS (PDF#96-153-3063).

Figure 4a,b show the experimental XRD patterns for the drop-cast CsPbBr_3_ thin film on the glass substrate depending on the surface ligand species: (Figure 4a) OA and OAm and (Figure 4b) OO and OAm, whereas Figure 4c,d shows the XRD patterns for (Figure 4c) cubic and (Figure 4d) orthorhombic based on Joint Committee on Powder Diffraction Standards (JCPDS). As shown in Figure 4a–c, the location of XRD peaks such as (100), (110), and (200) are more corresponding to that of cubic (JCPDS: PDF#96-153-0682: space group $Pm\bar{3}m$) instead of orthorhombic in spite that it was synthesized at RT [29,55,72,73]. Note that the lattice constant is 5.6050 Å based on the above JCPDS data, which was confirmed by the *d*-spacing of ~5.478 Å or 5.418 Å = λ/(2⋅sinθ) at *θ* = 8.08°(OA) or 8.17° (OO), respectively, when the X-ray wavelength (*λ*) is 1.5406 Å. Probably, the CsPbBr_3_ NC may prefer to exist in cubic phase due to its high surface energy and capping ligand effects. Furthermore, all the drop-cast CsPbBr_3_ nanocrystalline thin films on the glass substrate display two intense peaks at 2*θ* = 16° and 32°, corresponding to (100) and (200) crystallographic planes, respectively, indicating that there is orientational order to the (100) direction. However, when the OO and OAm ligands were incorporated as a ligand system for CsPbBr_3_, the additional (110) peak is clearly observed, indicating that the mixed ligand system by OO’s glycerol fraction makes the orientational order be reduced, resulting in a partial amorphous halo (see the baseline shape centered around (110) peak). However, when we estimated the crystallite size (*t*) by Scherrer equation (Equation (3)), it was in the range of ~40–42 nm for all the samples (Table 2).

(3) $$t=\frac{0.9\cdot \lambda }{B\cdot \cos\theta }$$

where *λ* (= 0.154 nm) is the X-ray wavelength, and *B* is the full width at half maximum (FWHM) at the angle *θ*. Here, it is noteworthy is that the estimated crystallite size is a direct particle size of this single crystal in nanoscale, which will be further addressed in the below section.

**Figure 5:**
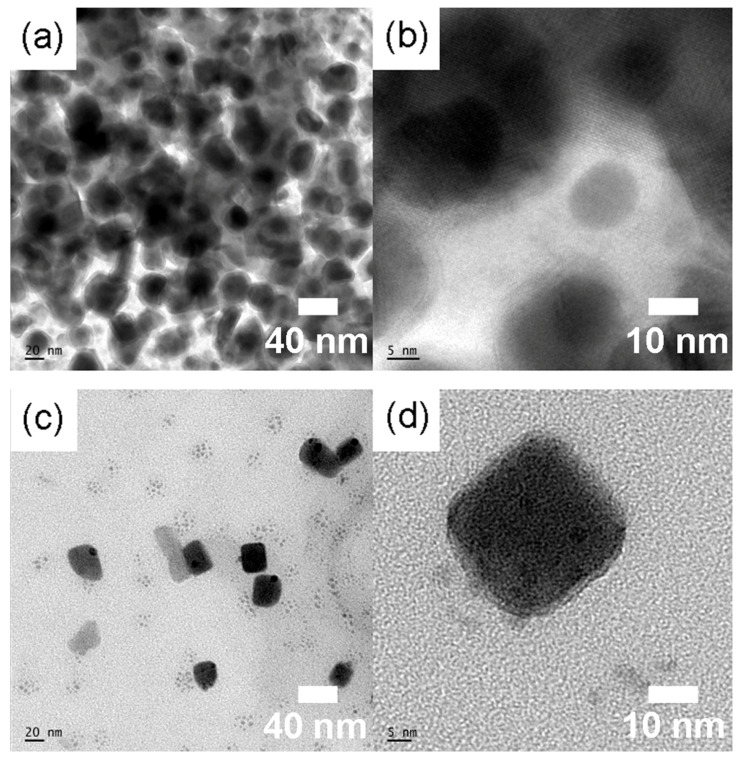
HR-TEM images of CsPbBr_3_ nanocrystals (nano cubic) when the ligand was oleic acid and oleylamine (**a**,**b**) and olive oil and oleylamine (**c**,**d**).

Figure 5 shows HR-TEM images for CsPbBr_3_ depending on the surface ligand system. Figure 5a,b show the case of OA and OAm, whereas Figure 5c,d show the case of OO and OAm. As shown in Figure 5, the TEM images show roughly a nano cubic, which is in line with the XRD data exhibiting the cubic structure.

**Figure 6:**
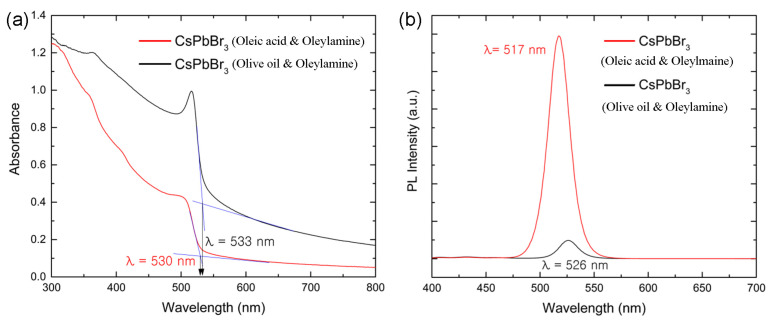
(**a**) UV-Vis absorbance spectra of the CsPbBr_3_ nanocrystal dispersion depending on the ligand species. Here, *λ* = 530 nm corresponds to *E_g_* ≈ 2.34 eV (oleic acid and oleylamine) and *λ* = 533 nm corresponds to *E_g_* ≈ 2.33 eV (olive oil and oleylamine). (**b**) PL spectra of CsPbBr_3_ nanocrystals depending on the ligand species.

Figure 6 shows (a) the UV-vis absorption and (b) the PL emission spectra for the two different colloidal dispersions containing CsPbBr_3_ NCs depending on the ligand species. Here, it is noteworthy that the CsPbBr_3_ perovskite is a direct semiconductor. In addition, the synthesized CsPbBr_3_ NCs do not display any grain boundary in Figure 5, indicating a single crystalline nature in nanoscale although there might be some surface defects. Therefore, the CsPbBr_3_ NCs are different from the disordered conjugated polymers showing a localized state. For disordered system, Mott defined a mobility edge between localized and delocalized states [74]. However, in the case of CsPbBr_3_ NCs, the sharp absorption edge is expected, affording the decision of optical bandgap by using a simple tangential slope based on the equation, E=hc/λ, where *E* is energy, *h* is Planck’s constant, *c* is the speed of light, and *λ* is the wavelength of light. Therefore, according to the UV-Vis absorption edge (530 nm vs. 533 nm) in Figure 6a, the corresponding optical bandgap (*E_g_*) is 2.34 eV = 1240/530 (for OA and OAm) and 2.33 eV = 1240/533 (for OO and OAm), respectively. See Equation (4) for the conversion factor, 1240, between the energy (bandgap) and the wavelength (absorption edge).

(4) $$\lambda \left[\mathrm{nm}\right]=\frac{hc}{E}=\frac{\left(6.62607\times 10^{-34}\;J\times s\right)\times \left(3\times 10^{8}\;m/s\right)\left(10^{-9}\;\mathrm{nm}/1\;m\right)}{E_{g}\left(\mathrm{eV}\right)\times \left(1.60218\times 10^{-19}\;J/1\;\mathrm{eV}\right)}=1240\frac{1}{E\left[\mathrm{eV}\right]}$$

This result indicates that the nanoparticle size might be slightly larger in CsPbBr_3_ synthesized with OO and OAm. This result is in line with the estimated crystallite size trend according to the sharpest peak at the (200) crystallographic plane in Figure 4a and Table 2. On the other hand, the PL spectra exhibit the PL peak at 517 nm for CsPbBr_3_ with oleic acid and oleylamine and at 526 nm for CsPbBr_3_ with OO and OAm. Here, the shift of PL peak from 517 nm to 526 nm (a red shift) indicates that the effective crystal size (or aggregation) of CsPbBr_3_ NCs is slightly larger in the colloidal dispersion state when OO and OAm were employed as the surface ligands, although the apparent size (Table 2) is similar for both ligand systems in the solid state. Furthermore, it is believed that the surface state of NCs is different depending on the NC–ligand complexations. Hence, when OO and OAm were complexed with CsPbBr_3_ NCs, there might be more surface defects, resulting in more non-radiative recombination (i.e., smaller PL intensity) in Figure 6b. Furthermore, FWHM is 21 nm for the former, but 19 nm for the latter. According to our finding, the narrow emission spectra were obtained below 25 nm, which falls in the required range of 12–42 nm [51] for the optoelectronic applications such as lighting and display technology.

**Figure 7:**
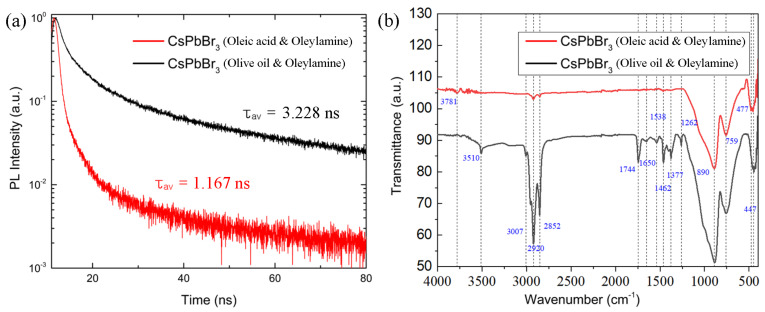
(**a**) PL spectra decay of CsPbBr_3_ nanocrystals depending on the ligand species. (**b**) FT-IR spectra of CsPbBr_3_ nanocrystals depending on the ligand species.

Figure 7a shows PL decay curve for the colloidal CsPbBr_3_ NC dispersion as a function of ligand species. The average lifetime of electron–hole pairs (Wannier–Mott excitons) is 1.167 ns for CsPbBr_3_ with OA and OAm, whereas it is 3.228 ns for CsPbBr_3_ with OO and OAm, indicating that a slightly larger CsPbBr_3_ with OO and OAm displays a longer life time of excitons. Figure 7b shows the FT-IR spectra for the drop-cast CsPbBr_3_ nanocrystalline thin film. First of all, the IR spectrum for CsPbBr_3_ with OA and OAm is relatively simpler than that for CsPbBr_3_ with OO and OAm. Second, the FT-IR spectrum for OO and OAm shows a high absorption at 3020–2850 cm^−1^, whereas the corresponding spectrum for OA and OAm is very weak at those wavenumbers, which correspond to the symmetric and asymmetric C-H stretching modes [53,54]. The functional groups of other organic components can be attributed to the absorption peaks at 3781 cm^−1^ [54] and 3510 cm^−1^ [53], which originate from the N-H characteristic absorption. The IR peaks at around 1800–1600 cm^−1^ are related to carbonyl (C=O) mode of stretching vibration which can be shift to lower wavenumber due to any perturbation. The shift of wavenumber may be ascribed to the presence of hydrogen bonding. The peaks at 1538 cm^−1^ and 1462 cm^−1^ are related to C-CH_3_ asymmetric bending. On the other hand, the peaks at 1377 cm^−1^ and 1262 cm^−1^ correspond to C-CH_3_ symmetric bending vibration and C-N stretching vibration, respectively. Absorption peaks in the range of 1000–500 cm^−1^ indicate that the existence of aromatic bending vibrations [75], which might originate from OO components, trapped toluene (C_6_H_5_CH_3_), and/or aromatic impurities in samples. The peak at 477–447 cm^−1^ is associated with CsPbBr_3_–OAm complexes [76].

## 4. Conclusions

In this work, we demonstrated the usefulness of olive oil and oleylamine as the surface ligand system (vs. the typical oleic acid and oleylamine mixtures) for the synthesis of CsPbBr_3_ nanocrystals at room temperature. For this purpose, the supersaturated recrystallization process was employed, enabling a direct synthesis of perovskite nanocrystals. First, according to XRD, the crystallite size is ~40–42 nm in both conditions (oleic acid and oleylamine vs. olive oil and oleylamine), whereas the latter system allows for a less orientational order than the former by showing a partial amorphous halo. The TEM images confirmed that the CsPbBr_3_ nanocrystals have a cuboid shape in line with XRD data exhibiting a cubic phase. The UV-Vis absorption data exhibited that the optical bandgap of CsPbBr_3_ is about 2.3 eV in both ligand systems. The PL emission spectra indicated that FWHM is about 19 nm (olive oil and oleylamine) vs. 21 nm (oleic acid and oleylamine), which falls into the desirable FWHM values, i.e., ~12–42 nm for technical applications. Finally, the average PL lifetime is 3.228 ns for olive oil and oleylamine and 1.167 ns for oleic acid and oleylamine. Future work may include the application of CsPbBr_3_ nanocrystals to the biosensors and optoelectronic devices.

## Figures and Tables

**Table 1:** Gutmann’s solvent donor number (*D_N_*), solubility parameter (δ), dielectric constant (ε), molar mass, density, and boiling point (b.p.), and the chemical structures of solvents.

Solvent	*D_N_* (kcal/mol)	*δ* ^1^ (cal/cm^3^)^1/2^	ε	Molar Mass (g/mol)	Density (g/cm^3^)	b.p. (°C)	Chemical Structure
DMF	26.6	12.1	36.1	70.095	0.948	153	C_3_H_7_NO
Toluene	0.1	8.9	2.38	92.140	0.867	111	C₆H₅CH₃

^1^ In MKS unit, the *δ* value is 24. 8 MPa^1/2^ for DMF and 18.2 MPa^1/2^ for toluene, respectively.

**Table 2:** Crystallite size (*t*) of CsPbBr_3_ at the (200) crystallographic plane. Here, β denotes a full width at half maximum (FWHM).

	CsPbBr_3_ Nanocrystals				
	Crystal Planes	2*θ* (°)	*θ* (°)	*β* (Radian)	*t* (nm)
Oleic Acid (OA)	(100)	16.15	8.08	0.003323	42
	(200)	31.65	15.83	0.003641	40
Olive Oil (OO)	(100)	16.33	8.17	0.003438	41
	(200)	31.80	15.90	0.003438	42

## Data Availability

The datasets used and/or analyzed during the current study are available from the corresponding author upon reasonable request.

## Supplementary Materials

The following are available online at: https://www.mdpi.com/article/10.3390/mi14071332/s1, Figure S1: The electronic structures of CsPbBr_3_ unit cells displaying polymorphism: (a) orthorhombic, (b) tetragonal, and (c) cubic phase. Here, the bandgap energy (*E_g_*) of each unit cell is 2.31 eV for orthorhombic, 2.37 eV for tetragonal, and 2.40 eV for cubic phase, respectively. Figure S2: Vials containing a CsPbBr_3_ colloidal dispersion depending on surface ligands: (a) oleic acid and oleylamine—a normal photo, (b) oleic acid and oleylamine—under 365 nm UV light, (c) olive oil and oleylamine—a normal photo, and (d) olive oil and oleylamine—under 365 nm UV light. Hence, CsPbBr_3_ nanocrystals can serve as a green-light emitter.
