# Supplementary material for: All-Inorganic CsPbBr3 Perovskite Nanocrystals Synthesized with Olive Oil and Oleylamine at Room Temperature
Source: Micromachines (Basel). 2023 Jun 29;14(7):1332. doi: 10.3390/mi14071332 (PMC10383321; doi:10.3390/mi14071332)
Supplement: Supplementary file 1 [file micromachines-14-01332-s001.zip › micromachines-2470409-supplementary.pdf]

micromachines

*Supplementary Materials*

# All-Inorganic $CsPbBr_3$ Perovskite Nanocrystals Synthesized with Olive Oil and Oleylamine at Room Temperature

**Getachew Welyab [1,2], Mulualem Abebe [1], Dhakshnamoorthy Mani [1], Aparna Thankappan [3], Sabu Thomas [4], Fekadu Gochole Aga [5,6] and Jung Yong Kim [5,6,*]**

1 Faculty of Materials Science and Engineering, Jimma Institute of Technology, Jimma University, Jimma P.O. Box 378, Ethiopia; gwelyabt@gmail.com (G.W.); muabeme@gmail.com (M.A.); m.dhakshnamoorthy@gmail.com (D.M.)
2 Department of Physics, College of Natural and Computational Science, Mizan-Tepi University, Mizan P.O. Box 260, Ethiopia
3 Department of Physics, Baselius College, Kottayam 686001, India; aparnathankappan@baselius.ac.in
4 School of Energy Materials, Mahatma Gandhi University, Kottayam 686560, India; sabuthomas@mgu.ac.in
5 Department of Materials Science and Engineering, Adama Science and Technology University, Adama P.O. Box 1888, Ethiopia; fekadu.gochole@astu.edu.et
6 Center of Advanced Materials Science and Engineering, Adama Science and Technology University, Adama P.O. Box 1888, Ethiopia
* Correspondence: jungyong.kim@astu.edu.et

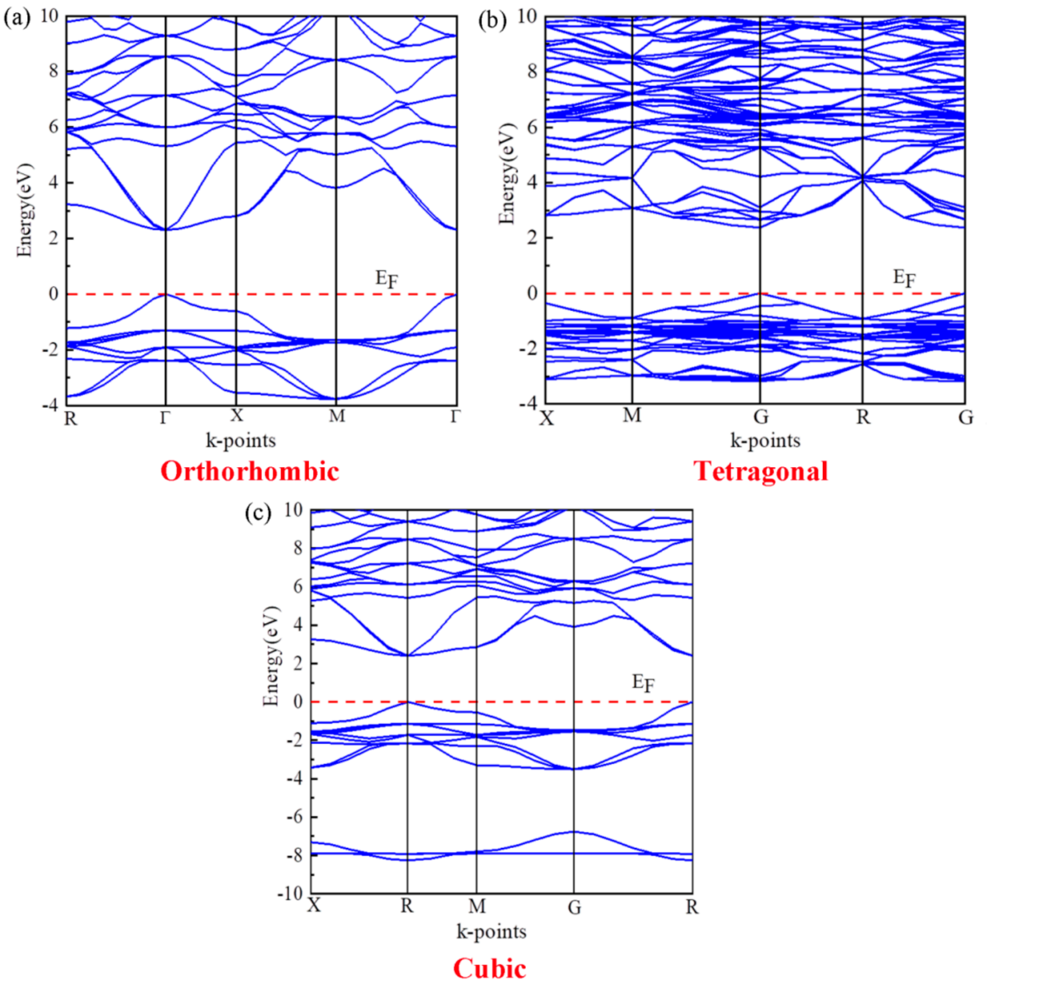


**Figure S1.** The electronic structures of $CsPbBr_3$ unit cells displaying polymorphism: (**a**) Orthorhombic, (**b**) tetragonal, and (**c**) cubic phase. Here, the bandgap energy ($E_g$) of each *unit cell* is 2.31eV for orthorhombic, 2.37 eV for tetragonal, and 2.40 eV for cubic phase, respectively.

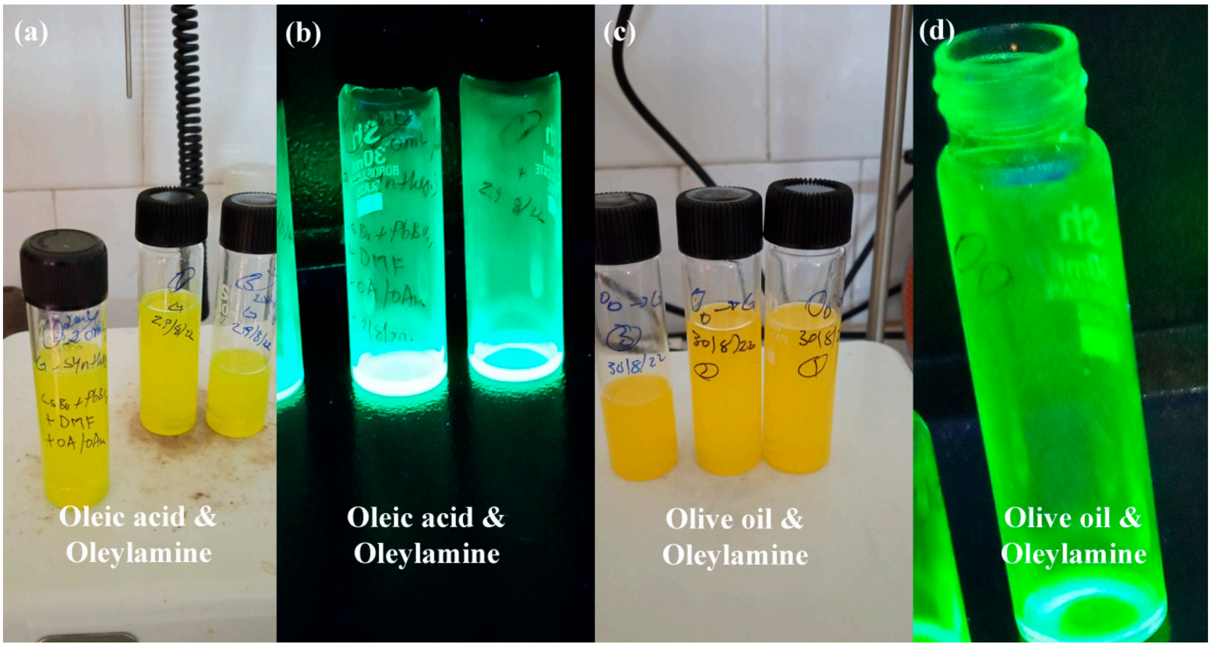


**Figure S2.** Vials containing a $CsPbBr_3$ colloidal dispersion depending on surface ligands: (**a**) oleic acid and oleylamine–a normal photo, (**c**) oleic acid and oleylamine–under 365 nm UV light, (**c**) olive oil and oleylamine–a normal photo, and (**d**) olive oil and oleylamine–under 365 nm UV light. Hence, $CsPbBr_3$ nanocrystals can serve as a green-light emitter.
